# Automated news in practice: a cross-national exploratory study

**DOI:** 10.12688/openreseurope.16040.2

**Published:** 2023-09-06

**Authors:** Samuel Danzon-Chambaud

**Affiliations:** 1 School of Communications (PhD graduate), Dublin City University, Dublin, Ireland

**Keywords:** automated news, automated journalism, algorithmic journalism, computational journalism, news production, actor-network theory

## Abstract

**Background:** This article provides a comprehensive picture of the current state of automated news—understood here as the auto-generation of journalistic text through software and algorithms—as well as to where it is headed. For this, I look at 18 news organisations in Europe, North America and Australia, following a strategic sample inspired by Hallin and Mancini’s (2004) media system typology.

**Methods:** To conduct this cross-national exploratory study, I made use of semi-structured interviews with editorial staff, executives and technologists. I also rely on Actor-network theory (ANT) to tell when an *interference* is made to an otherwise *linear* situation, thus endowing automated news with a sense of agency.

**Results:** Overall, my findings show that the main interferences concern alternate data sources (e.g., news organisations’ internal feeds, crowdsourced material), in-house interfaces that allow for more journalistic participation (e.g., internal self-editing tools, notification streams) and output other than text (e.g., automated audio summaries for voice assistants).

**Conclusions**: Although these changes lead to greater journalistic professionalisation, they could also make news organisations become too dependent on Big Tech companies for data acquisition and dissemination of automated news products. That said, mutual negotiations and a re-alignment of interests may occur as platforms increasingly face journalistic challenges.

## Introduction

In recent years much attention has been given to automated news, a computer process generally understood as the auto-generation of journalistic text through software and algorithms, with no human intervention in-between except for the initial programming ( Carlson, 2015; Graefe, 2016). Automated news—which is also sometimes referred to as “automated journalism”, “algorithmic journalism” or “robot journalism”—relies on a basic utilisation of Natural Language Generation (i.e., NLG), a computer technique that has been used for several decades to generate text in areas like sports, finances and weather forecasting ( Dörr, 2016). In the case of automated news, NLG algorithms are used to fetch information on external or internal datasets, this in order to fill in the blanks left on pre-written text. This resembles a bit the game “Mad Libs” ( Diakopoulos, 2019), as programmers or editorial staff need to come up with templates that, on the one hand, include enough elements that can be predicted in advance and, on the other hand, can be connected to a big enough data flow.

Because of these limitations, only a small range of stories can be automated this way, for instance election results, financial news or sports summaries. Although there is little machine learning involved at the moment, this is becoming a growing area of interest: some machine learning applications of NLG production are already being advertised on the websites of companies that specialise in delivering automated content to business, media, and governmental organisations alike ( Narrativa, no date 1); the European Union-funded project EMBEDDIA is looking at including elements of machine learning in automated news generated using pre-written templates to make it less formulaic and nicer to read (see Leppänen, 2023; Rämö & Leppänen, 2021); and the Czech news agency ČTK has been experimenting with machine learning techniques to generate automated news templates, with the help of a research team at the University of West Bohemia ( Stefanikova, 2019). Recent breakthroughs related to large language models (e.g., ChatGPT, Google’s Bard) have certainly stirred up debate, yet for the moment there is reason to believe that their opacity and unreliability do not make for journalistic usage ^1^.

Automated news started to be more discussed in the 2010s as *The Los Angeles Times* began covering homicides in an automated fashion ( Young & Hermida, 2015) and launched a tool to generate earthquake alerts ( Schwencke, 2014), while The Associated Press partnered with the firm Automated Insights to automate corporate earnings stories ( Colford, 2014). Proponents of automated news typically develop their technology in-house, outsource it to an external content provider or use third-party solutions that let journalists design their own automated stories. For instance, the *Washington Post* developed an in-house tool to produce short automated pieces during the 2016 Rio Olympics ( WashPost PR Blog, 2016a); *Le Monde* collaborated with the firm Syllabs to automatically cover the results of the 2015 regional elections in France ( Rédaction du Monde.fr, 2015); and the BBC subscribed to an online platform, Arria NLG Studio, that lets journalists template out their own automated stories using a type of No-code language that makes it accessible to editorial staff with little computing experience ( Molumby & Whitwell, 2019). As for its types of usage, automated news can be used to publish simultaneously at scale, as the Swiss media group Tamedia did with the generation of almost 40,000 hyperlocal stories to report on the outcome of a referendum ( Plattner & Orel, 2019), or serve as first drafts to assist journalists with their own writing, as this seems to be the case at *Forbes* and at the *Wall Street Journal* ( Willens, 2019; Zeisler & Schmidt, 2021).

 In this article extracted from my PhD thesis ( Danzon-Chambaud, 2023) ^2^, I will provide a more complete picture of the use of automated news, conducting a cross-national exploratory study for this. I will rely on Actor-network theory (ANT) concepts to distinguish when an *interference* is made to otherwise *linear* situations where initial intent is kept and where it does what it is supposed to do. My sample for this research is made of 18 organisations based in 11 countries, which are representative of three media types (i.e., public service broadcasters, newspapers, news agencies); it follows a sampling strategy inspired by Hallin and Mancini’s (2004) media system typology as I looked at newsrooms that belong to the Mediterranean, North/Central and North Atlantic models. Due to COVID-19 limitations, I have made use of semi-structured interviews that were conducted remotely between September 2020 and April 2021, with email exchanges taking place up until 2022 to make sure content remained current and accurate. Other elements like screenshots and online material were also analysed in complement to these research interviews.

### Algorithms in news production

To begin with, I will provide a brief overview of recent developments in the use of algorithms for news production. The first has to do with data mining techniques. Diakopoulos (2019) illustrates how advanced machine learning models—in other words the use of algorithms to make statistical inferences or classifications based on a large corpus of data—were used in investigative journalism to retrieve newsworthy material off a massive amount of documents. He cites the work of *The Atlanta Journal Constitution*, which managed to expose 2,400 doctors that have been disciplined for sexual misconduct in the United States while examining more than 100,000 records, using for that an algorithm that scored and sorted through documents based on the likelihood that an abuse had, indeed, actually occurred. That said, Stray (2019) highlighted how the use of such models in investigative journalism also comes with its own set of issues, like high error margins that need to be compensated with estimates, difficulties in accessing the data in the first place, or the high costs of deploying such models just for a one-off project. However, collaborative efforts in investigative journalism today could help solve some of these issues, as shown by the growing use of machine learning by the International Consortium of Investigative Journalists, in investigations like the “Implant Files”, the “Mauritius Leaks”, the “Luanda Leaks” or the “Pandora Papers” ( Díaz-Struck *et al.,* 2020; Díaz-Struck *et al.*, 2021; Walker Guevara, 2019; Woodman, 2019). 

Another manifestation of algorithmic news production can be found in initiatives that aim at automating fact-checking (see Danzon-Chambaud, 2020). Diakopoulos (2019) identifies similar machine learning methods where a trained algorithmic model can be deployed on textual data so as to reveal claims that are worth fact-checking. This is for instance what Duke University Reporters' Lab has been doing to verify some of CNN’s transcripts with a software called “ClaimBuster”. Diakopoulos also writes about more basic methods that consist in matching textual data against a database made of previous fact-checks and reliable data, as in the British charity Full Fact’ efforts to debunk false claims in screen captions. In his report for the Reuters Institute for the Study of Journalism, Graves (2018) essentially details the same two approaches while putting a special emphasis on *stance detection*, a machine learning technique that tries to figure out whether a claim is supported or not.

In terms of algorithmic news production, though, automated news is by far what has got the most media and scholarly attention. Leaving aside the fret caused by the use of the term “robot journalism”—which, again, is inaccurate since it is only software and has no mechanical part (see Lindén & Dierickx, 2019)—automated news is generally talked about in two complementary ways: to compare its performance against real, human-written text and to assess its potential impacts on the labour market. In a systematic literature review I conducted on automated news research ( Danzon-Chambaud, 2021a), I found that scholarship on readers’ perceptions evaluated it to be very close to human-written text, with the exception of reading for pleasure (see also Graefe & Bohlken, 2020). Yet, as far as studies on practice were concerned I was unable to formulate a similarly cohesive and insightful argument. Most of the time automated news is viewed as a rather “passive” actor that, at best, helps journalists with routine tasks while they focus on more demanding work or, at worst, plays the role of a cold technology about to supplant them. I believe there is a fine line between these two, and am therefore interested in the following research questions:

RQ1. What results do we yield when we give agency to automated news?

RQ2. What potential implications does this bear for journalism practice and for journalism as a whole?

## Methods

To answer these questions, I will conduct an exploratory study that spans across groups of countries, so as to have a diverse range of news organisations included in this research. I have chosen a sampling strategy inspired by Hallin and Mancini’s (2004), which has often been used as a guiding framework to draw cross-national strategic samples of news organisations (see Cornia *et al.*, 2019; Cornia *et al.*, 2020; Menke *et al.*, 2018; Sehl *et al.*, 2019; Sehl & Cornia, 2021; Sehl *et al.*, 2021; Van den Bulck & Moe, 2018) Hallin and Mancini distinguish three types of media systems, based on their analysis of a set of dimensions that range from the structure of media markets to professionalisation and the role of State. These are: the “Mediterranean” or “Polarised Pluralist Model”, which includes countries such as France, Spain and Italy and is characterised, among others, by a low level of journalistic professionalisation—not dissimilar to political activism—and by strong connections with the State given delayed liberalisation in these countries (even if commercial influences have progressively grown in importance); the “North/Central European” or “Democratic Corporatist Model”, which concerns countries like the Nordics, Germany and Switzerland and where the media are considered social institutions that need to be protected by the State due to the pluralistic and consensus-based nature of these democracies, but still have a high degree of commercialisation and journalistic professionalisation; and the “North Atlantic'' or “Liberal Model”, which extends to countries like Canada, the United States and the United Kingdom, where commercialisation and journalistic professionalisation are relatively high and the role of the State moderated, even if sometimes commercial influences can circumscribe journalistic independence.

As for choosing media types, I have decided to include news agencies, newspapers and public service media for the following reasons: first, the use of automated news at news agencies is well established, especially in Europe (see Fanta, 2017); second, it can be argued that newspapers are more likely to engage with this form of technology, as their business model that is under threat because of the digital turn forces them to be more innovative, as opposed for instance to commercial broadcasters that can still rely on stable advertising revenues and on other types of incomes ( Cornia *et al.*, 2019); third, public service media can be considered leaders in providing “thorough” data journalism pieces to audiences (see Borges-Rey, 2016; De Maeyer *et al.*, 2015), especially as data journalism experts are more likely to be hired at public service broadcasters in Germany ( Beiler *et al.*, 2020) and as public service media in Australia developed their own in-house solutions ( de-Lima-Santos *et al.*, 2021): this can let us posit that the kind of programming skills that is at use in data scraping activities can also be leveraged to set up automated news.

For this exploratory study, I have relied on purposive sampling to select 18 news organisations, with each pair representing a different combination of media types and media systems (see Table 1). Due to COVID-19 limitations, semi-structured interviews with editorial staff, executives and technologists were conducted remotely between September 2020 and April 2021 (see Appendix A), with email exchanges taking place up until 2022 to make sure content remained current and accurate. The questions I asked generally followed the same structure (see the example of the questionnaire used with the *Washington Post* under “data availability”), while still retaining a degree of flexibility to adapt to the interviewee’s or the organisation’s specifics. To do this type of study, I obtained approval from my university’s research ethics committee. Among these interviewees were 8 BBC staffers that I could gain access to thanks to a secondment I did with my research program. Interviewees were contacted by email or *via* social media, a gatekeeper’s approval being sometimes needed ^3^. Their names were not divulged so that they could speak more freely, although it is most likely that their hierarchy knew that they were participating in this research project. Written informed consent was obtained from each of the participants, and they were given the opportunity to review some of their statements that dealt with potentially sensitive or unclear information, but not my own interpretation over what they shared. My exchanges were rather smooth, my interviewees generally knowing what I was asking about and not being caught off-guard (they were given an indication of what will be discussed, but were not handed the interview questions in advance). I asked for clarifications in follow-up emails when needed. Finally, sex and gender were not considered to be particularly relevant in this study: as such, interviewees were not asked to disclose their gender, but in a strictly binary sense it turned out that 23 of them were male and 5 were female, thus reflecting a gender gap that could be further investigated.

**Table 1.** News organisations studied based on media systems and media types ^4^.

Media systems	News agencies	Newspapers	Public broadcasters
North Atlantic	Associated Press (United States) Reuters (United Kingdom)	Washington Post (United States) The Times (United Kingdom)	BBC (United Kingdom) ABC (Australia)
North/Central	STT (Finland) NTB (Norway)	Stuttgarter Zeitung (Germany) Tamedia (Switzerland)	YLE (Finland) Bayerische Rundfunk (Germany)
Mediterranean	AFP (France) ANSA (Italy)	El Confidential (Spain) Rossel/Sudpresse (Belgium/France)	France Bleu (France) RTVE (Spain)

I also analysed material published online (e.g., blog posts, trade publications, etc.) so as to have a better overview of the way automated journalism is implemented: these are linked to or referenced as such in my findings section; otherwise, information comes from statements collected over the course of my interviews. In addition, screenshots of automated news software or material that was found online or forwarded to me by research participants are also featured there. These elements are essentially informative, as they were used to complement my research interviews and to verify what my interviewees have said.

## ANT analytical framework

To give agency to automated news and no longer see it as a “passive” technological artefact, I will make use of concepts that belong to Actor-network theory (ANT), which I will briefly outline here. A fundamental aspect of ANT is that it essentially revisits sociology using a “bottom-up” perspective and rejects more traditional “top-down” frameworks. Instead, it encourages the researcher to follow, from scratch, a “rich bestiary of significant actors” ( Clark, 2020, p. 160)—or rather *actants* ( Blok, 2019; Crawford, 2005)—which involves *human* and *non-human* elements that can be as diverse as ( Michael, 2017a, p. 5) “mundane objects, exotic technologies, texts of all sorts, nonhuman environments and animals”. The term “actor-network” in itself speaks to the idea that every actor and all of its attributes—such as thinking, writing or loving for humans—are never entirely cut out from each other, thereby creating a “web of relations” that stretches “both within and beyond the body” and across which force or effect is distributed ( Law, 1992, p. 384; Primo, 2019, p. 2). To better understand this, Law uses the following metaphor about himself ( 1992, pp. 383–384): “If you took away my computer, my colleagues, my office, my books, my desk, my telephone I wouldn't be a sociologist writing papers, delivering lectures, and producing "knowledge." I'd be something quite other—and the same is true for all of us.” As such, ANT is therefore well suited to studying change in practice ( Plesner, 2009); in the case of journalism, it helps account for all the “tools of the trade” (e.g., web searches, databases, smartphones) that make it as it is today, and can be used to document journalistic innovation, including automated news ( Primo, 2019, p. 2).

Another marker of ANT is the concept of *translation*: not to be confused with language translation, ANT’s *translations* rather speak to a phenomenon whereby *heterogeneous* entities ( Law, 1984) or *actants* come together to form an actor-network while re-aligning their interests, thus potentially disengaging themselves from other structures they belong to ^5^. In his study depicting how a scallop species ( *Pecten Maximus*), local fishermen, the scientific community and a team of biologists engaged into forming an actor-network whose goal is to replenish scallops beds in the Saint-Brieuc bay, in France, Callon (1984) described how each of these entities *translated* their interests (in several steps, though not entirely detailed here) so that they pass through an *obligatory point of passage*—a common objective—which makes the network hold; in this case, it is the biologists’ novel research programme, who then become *spokespersons* for the group. As Callon ( *ibid.*, p. 224) put it: “Translation is the mechanism by which the social and natural worlds progressively take form. The result is a situation in which certain entities control others.” That being said, for the actor-network to be able to last in time, successful *enrolment*, or ( *ibid.*, p. 211) “the device by which a set of interrelated roles is defined and attributed to actors who accept them”, needs to be sustained, making it a structure where relational power is always up for negotiations ( Michael, 2017a). If robust enough, though, it may give rise to a *macro actor* that is able to restructure society as whole ( Cooren, 2009; Czarniawska, 2016).

In ANT, the process through which entities undergo *translations* and become stabilised enough to form an actor-network comes under terms like *simplification*, *black-boxing* or *punctualisation* ( Crawford, 2005; Michael, 2017a). In addition, they can transport force or effect in either two ways: as an *intermediary* where meaning is maintained and where outputs can be predicted by inputs, and as a *mediator* where meaning is changed and where inputs are never a good predicator of outputs ( Latour, 2005). To explain these specifics, Latour gives the following example:

- A properly functioning computer could be taken as a good case of a complicated intermediary while a banal conversation may become a terribly complex chain of mediators where passions, opinions, and attitudes bifurcate at every turn. But if it breaks down, a computer may turn into a horrendously complex mediator while a highly sophisticated panel during an academic conference may become a perfectly predictable and uneventful intermediary in rubber stamping a decision made elsewhere ( Latour, 2005, p. 39).

When encountered intermediaries can take the form of *immutable mobiles* that are able to act at a distance ( Law, 1984) as no force or effect is changed as it passes through them. Taken as *quasi-objects* or *tokens* they can even play a role in connecting *actants* and in cementing the network, as it is for instance the case with money, some technical artefacts or *inscriptions* when it concerns textual or graphical elements ( Crawford, 2005; Hassard & Alcadipani, 2010; Latour, 2005; Michael, 2017b; Nikolova, 2008). Drawing on Callon (1990) and Latour (1992), Sayes (2014, p. 138) specifies that, ultimately, “an intermediary is a placeholder in the sense in which it merely does what anything else in its position would do”. By contrast, he writes, “a mediator is something *more* than this”. In the case of non-human elements, for instance, it is “seen as adding something to a chain of interaction or an association”. Put differently, intermediaries transport force or effect in a *linear* way whereas mediator *interfere* with this linearity, yielding a result that actively contributes to shaping the socio-material world. When conducting an ANT analysis, Latour then recommends striving to see each entity as a potential mediator, and not simply as an intermediary: this, he argues ( 2005, p. 128), would “render the movement of the social visible to the reader”. 

In media and communication research, ANT can be used to investigate the introduction of a new technological artefact in a well-established network, especially as it is faced with resistance: indeed, as the new technology is being embedded into the actor-network with its own intended meaning, a series of mutual *translations* happens, resulting in new power relationships. For example, while studying the deployment of a Personal Digital Production system (i.e., PDP) at the BBC—which allowed editorial staff to film and edit videos on their own— Hemmingway (2005, p. 25) found that a “initial rejections of PDP came from those people on the network unable to organise resources until PDP operators were also internalised and socialised within the network”.

In parallel, ANT can be employed to establish power relationships between entities that already exist in the network, as in Schmitz Weiss and Domingo’s account of innovation in online newsrooms (2010). They observed ( *ibid.*, p. 1063) that “the obligatory point of passage of the production team as translator of journalistic needs into technological developments hindered opportunities for innovative ideas to flourish”, as “breaking news reporters felt their ideas were neglected, and web developers limited themselves to following instructions from the online editor”. According to them ( *ibid.*, p. 1068), this made online journalists feel “powerless in the decision-making process” while technologists viewed their colleagues’ needs as a lack of skills. In the case of Internet and digital technologies being brought into Greek newsrooms, Spyridou *et al.* (2013) looked at interactions and power relationships between well-established actors (i.e., journalists), new actors (i.e., technological tools, convergence and participation) and former *intermediaries* with an increasingly important role to play (i.e., audiences), in order to find out, among others, whether the dominant journalistic culture adapts to or contributes to shaping new technological affordances. They eventually conclude ( *ibid*, p. 93) that these changes “tend to get normalized or ‘rationalized’ through the values and norms of the dominant journalistic culture”.

Even though ANT has been recommended by Primo (2019) to study automated news I found no use of it in my systematic literature review, which analysed 33 empirically-oriented scholarly articles published between 2005 and mid-2020. Although this review was by no means exhaustive as other publications on ANT and automated journalism fell outside my search criteria ^6^, it was still representative of a research gap worth of our attention. ANT certainly comes with its own limitations (see Ryfe, 2022), such as being too oblivious of any overarching social order and loosing track of the bigger picture (see Benson, 2017; Couldry, 2020), but it can meanwhile be used to give a “bottom-up” account of how automated news directly contributes to shaping the world, thus yielding agency. Distinguishing *mediators* from *intermediaries* then appears all the more necessary.

## Results

As described in my methodology, I will conduct here a cross-national exploratory study using Hallin and Mancini’s media system typology (2004) to strategically select news organisations, limiting myself to news agencies, newspapers and public service broadcasters. When analysing how automated news is implemented within these organisations, I will make use of ANT concepts to see whether it transports force or effect in a *linear* manner (i.e., *intermediary*) or if there is an *interference* to it (i.e., *mediator*), thereby changing the status quo and actively contributing to shaping socio-material reality.

### Linear situations

First, based on some of the most prominent examples that are developed in the introduction, it can be said that there is a *linear* effect to automated news when it merely transmits data from authoritative sources (private or governmental), when there is no direct journalistic involvement except through the affordances already provided for by third-party tools and—for now—when text only is generated, sometimes with visualisations, thus amounting to having *inscriptions*. Such an assemblage can be observed at news organisations outsourcing automated news to external content providers, like at the Associated Press, where teams collaborate with firms like Automated Insights and Data Skrive to come up with templates so that these companies can automate corporate earnings stories and sports recaps ^7^, based on private data (see Colford, 2014). Likewise, Italy’s news agency ANSA publishes weather forecasts that are sometimes generated using automation and data provided by a weather forecast company, but also national and regional accounts of the spread of COVID-19, using public data collected through Narrativa’s COVID-19 tracker initiative ^8^ and put together by the firm Applied XL ( Narrativa, no date 2; Redazione ANSA, 2020). As for Spain’s national public service broadcaster RTVE, it collaborated with Narrativa to run trials on less watched football competitions in Spain using private data and also prepared for generating stories on election results in small municipalities based on government data ( Corral, 2021). This is similar to what the French public radio broadcaster France Bleu and French newspapers belonging to the Belgian media group Rossel (e.g., *La Voix du Nord, L’Union*) have been doing during recent elections in France with automated news generated by the firms Syllabs (France Bleu) and LabSense (Rossel), based on governmental data. Besides, the Belgian newspapers group Sudpresse (owned by Rossel) and LabSense also collaborated on automating amateur football games in Belgium, sourcing data from a sports association.

**Figure 1.**
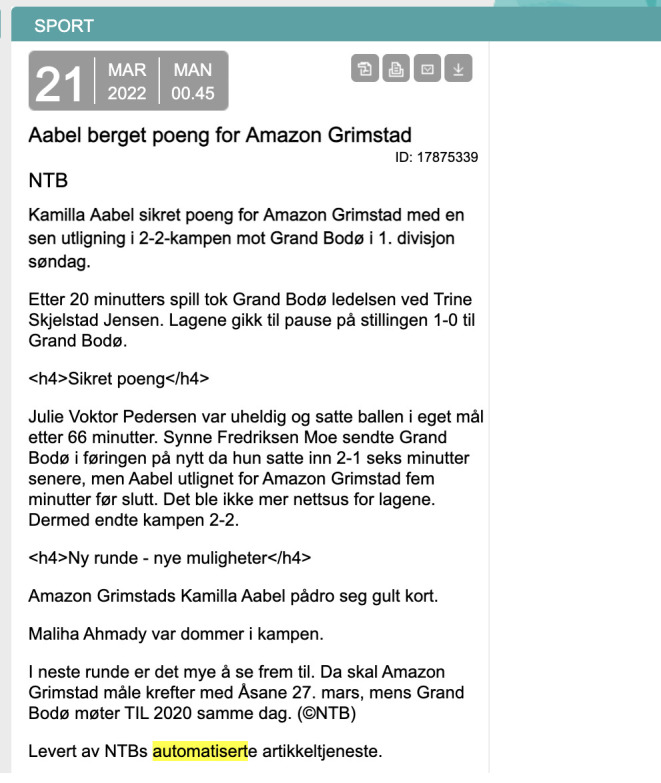
An example of an automated football game recap that was generated at NTB. This figure has been reproduced from NTB with permission, under a CC-BY NC 4.0 license.

A linear effect is also spottable when automated news is designed internally. As such, Reuters’ data team has been developing automated news the usual way while setting up stories on sports, financial news and COVID-19, relying both on private and public data. This was also true of *The Times*’ automated journalism project on COVID-19 (see Danzon-Chambaud, 2021b), which was based on public data and programmed in-house. As for the Norwegian news agency NTB, it relied on a select few editorial developers with both a journalistic and technical background so as to be able to automate the same type of pandemic-related content as well as sports, election and financial news (see Figure 1), using private and public data for this.

On their end, the data team at the newspaper *Stuttgarter Zeitung* programmed automated news to cover the 2021 German election at a municipal level with local governmental data, while a team of technologists at the Finnish public service broadcaster YLE developed automated summaries on sports and election results, using both private and public service data and helped by a journalist who can understand code. Moreover, YLE made its code for generating ice hockey recaps open source, following a Parliament's request to limit unfair competition in the Finnish media market: as a result, other organisations like Finland’s news agency STT used this code for their own ice hockey stories. Sometimes, an academic partner was also involved in the development of automated news, as in the Bavarian broadcaster Bayerischer Rundfunk’s collaboration with the Technical University of Munich to automate match reports for a basketball league in Germany ( Sebis Research News, 2021; Schneider & Köppen, 2021), which came in parallel with another project on COVID-19 (see Danzon-Chambaud, 2021b) and led to automating financial results as well ( Schneider, 2022). To do this, the team relied on public health sources for their COVID-19 project and on private data for sports and financial news. Lastly, after experimenting with their own solution to automate the Rio Summer Olympics, the 2016 presidential election in the United States and high school American football coverage ( WashPost PR Blog, 2016a; WashPost PR Blog, 2016b; WashPost PR Blog, 2017), the *Washington Post*’s engineering team joined forces with Northwestern University to develop a “computational political journalism R&D lab” ahead of the 2020 presidential elections ( Schmidt, 2019), thus improving existing automated news models that draw on data collected by private brokers during election time.

**Figure 2.**
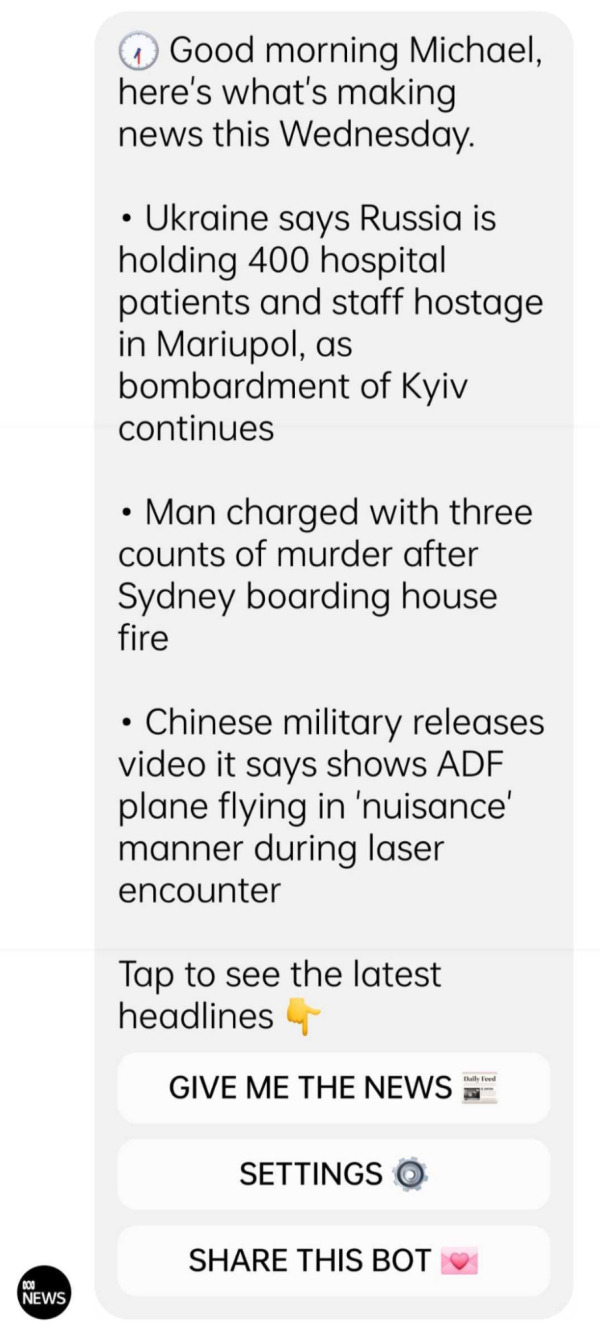
A daily news brief delivered by the Australian Broadcasting Corporation (ABC) through its conversational chatbot platform. This figure has been reproduced from ABC with permission, under a CC-BY NC 4.0 license.

A sense of linearity can also be found in the use of third-party self-editing tools that feature a form of No-code language, which allows editorial staff with little programming experience to design automated news on their own. This could be observed at the BBC, where the News Labs team used Arria NLG Studio to template out articles on A&E waiting times, tree planting and high street shopping, using public service datasets (see Danzon-Chambaud, 2021c). The Swiss newspaper group Tamedia used Wordsmith—Automated Insights’ own NLG technology that was made directly accessible to clients through a self-editing interface ( Mullin, 2015)—to draft out automated stories on referendums and election results in Switzerland ( Marchand, 2019; Plattner & Orel, 2019) and to provide a statistical roundup of the spread of COVID-19 (see Danzon-Chambaud, 2021b), using public service datasets. As for the Australian public service broadcaster ABC, it subscribed to a bot-building application, Chatfuel, to create a messenger bot (see Figure 2) that uses public service data to inform users on electoral results ( Archer, 2016; Elvery, 2016), but also to provide them with daily news summaries, weather forecasts and emergency alerts (see Ford & Hutchinson, 2019).

### Interferences 

In contrast this linear effect, mapping substantial *interferences* requires carefully thinking about what could potentially re-shape the “chain of interaction” or “association” ( Sayes, 2014) that automated news is part of. Thinking in a reverse way, this could be whenever these changes concern data other than coming straight from authoritative sources, systems that allow for direct journalistic participation other than through the affordances of third-party tools and, lastly, outputs other than text. First, with regards to other sources, a noticeable interference occurs as news organisations turn to their own internal feed, proceed to their own data collection or use archival material, thus avoiding the need to rely on third-party private or public service datasets. An example of this is the BBC’s and ABC’s efforts to connect their automated news system to an internal election results feed (see Danzon-Chambaud, 2021c for the BBC), which in the case of ABC is linked to the corporation’s own psephologist:

We're mostly looking at the data sources we use for broadcast to start with, or that are at that level. (...) The election one is coming from the Australian electoral commission or the State electoral commissions, but then it's going through our election expert's system, Antony Green. So it's being processed by his system and he's taking those raw figures and putting his knowledge of electoral systems over them to come up with predictions and things like that.
(Manager, ABC, Australia)

**Figure 3.**
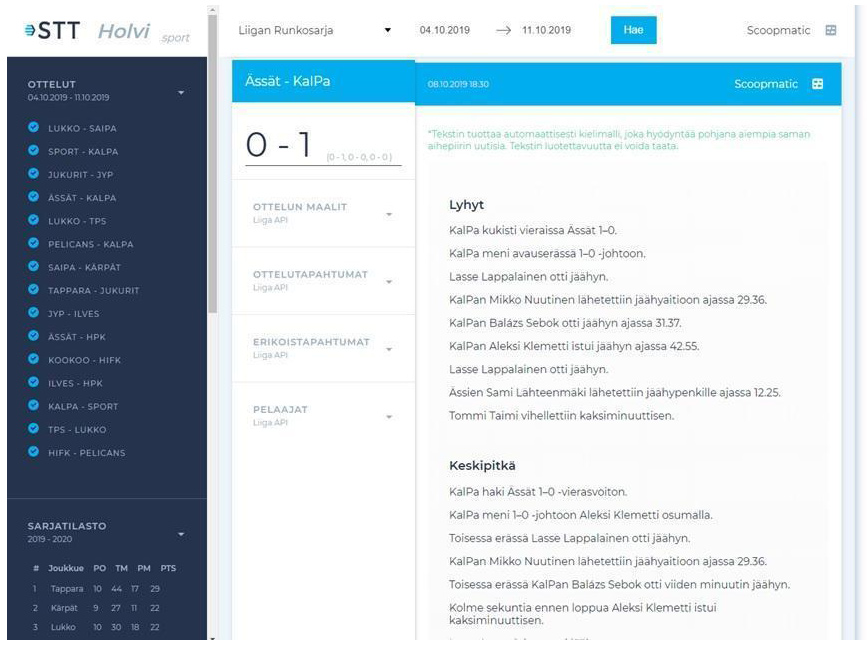
The interface on which ice hockey stories were generated using machine learning models that were trained on STT’s internal archives, which covered games that were held since the 1990s. This figure has been reproduced from STT with permission, under a CC-BY NC 4.0 license.

In a few instances, news organisations collected data on their own in order to automate news text, as shown in AFP’s and Reuters’ statistical roundups on the spread of COVID-19, which were both automated using shared spreadsheets that were manually filled by journalists on the ground, even if at Reuters this system was also connected to open data sources. As for tapping into archival material, the Finnish news agency STT collaborated for a time with the University of Turku, in Southern Finland, to automate ice hockey recaps using machine learning models ( Kanerva *et al.*, 2019) that were trained on STT’s own archives that dated back to the 1990s (see Figure 3). That being said, an executive at STT indicated that content generated this way did not meet the agency’s standards to be delivered to clients, but was accessible to them should they be interested in it:

It [STT’s archives] goes way back, but there wasn't enough reports for the AI because (...) it just wants all the data and more and more and more… And it wasn't enough for the AI to learn enough. And the second thing was that there was too much human… well, too much human in them. So there was, like, adjectives and things that weren't in the data that the machine was fed. (...) For example, in ice hockey there was, like, standings that weren't in the data that the machine was given. So we ended up using some manual work to go through, not all, but a lot of the stories.
(Executive, STT, Finland)

Additional interferences that related to source type were also visible in situations where crowdsourced material and social media feeds were used. The German newspaper *Stuttgarter Zeitung* relied on crowdsourced material to automate its air quality reports in the Stuttgart area, which were generated using AX Semantics’ self-editing tool and connected to open data sourced from a network of community sensors ( Plavec, 2017; Toporoff, 2017). In Australia, the ABC used opinion data collected through a polling exercise that is habitually done during election time so as to come with answers for its messenger bot ( Gee & Prior, 2018), an approach that was further extended to probe the public’s concerns on emergency preparedness. Social media feeds, on their end, were put to contribution using web scraping techniques, so as to be able to collect user-generated content and to conduct computational analysis on it. This was done, for instance, at the Spanish public service broadcaster RTVE, which partnered with the University Carlos III of Madrid to generate automated football stories that adopt a tone and voice that reflect users’ opinions ( del Rey García, 2020). “You can have the version for one team, for example: ‘It was a great success,’ the balanced news, and, on the other hand, (...) ‘they stole us the football match’”, said an executive at RTVE. Likewise, Reuters’ News Tracer acts as a “breaking news radar” while roaming on Twitter feeds to find relevant information, using advanced detection, classification and evaluation techniques for this; it then goes on to generate short automated text that is passed on to journalists for verification ( Emerging Technology from the arXiv, 2017; Liu *et al.*, 2017).

**Figure 4.**
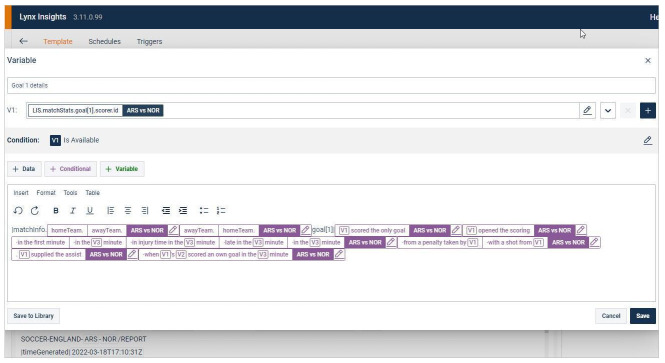
On Reuters’ Lynx Insight platform, journalists can template out their own automated stories using a form of No-code language that makes it accessible to news staff with little programming experience. This figure has been reproduced from Reuters with permission, under a CC-BY NC 4.0 license.

Another area where substantial interferences are brought into force relates to automated news systems specifically built for journalists and not limited to the affordances of third-party tools, thus allowing for additional tweaking. These can be, first, internal software that comes with its own self-editing tool, features notification streams and provides access to auto-generated background information. Reuters’ Lynx Insight system integrates all three: journalists can template out their own stories using a form of No-code language that resembles those of third-party tools (see Figure 4), receive Microsoft Teams notifications once stories generated this way (or that the data team set up) are ready and query the system as they look for automated news with background information. 

**Figure 5.**
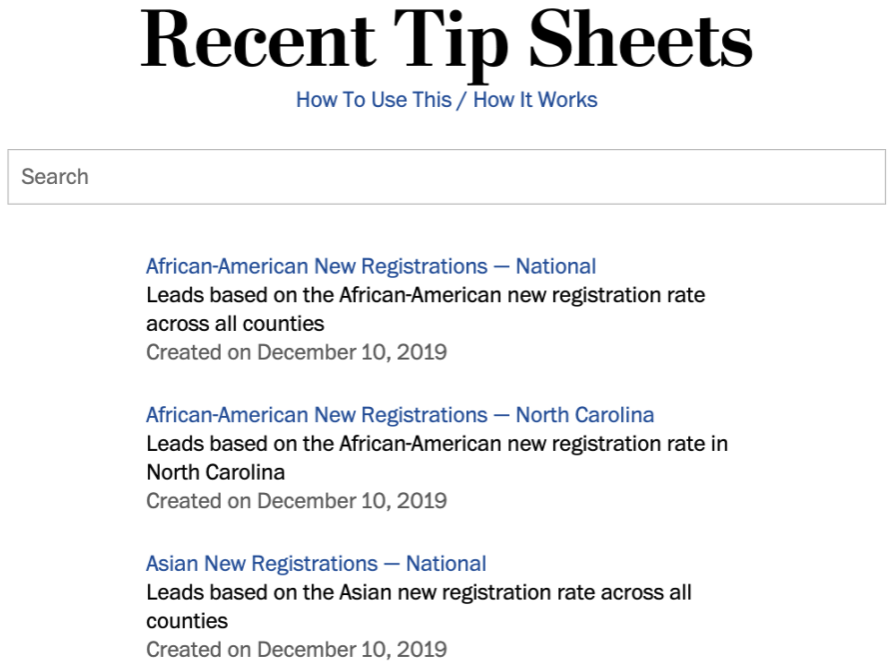
At the Washington Post, a query system was set up in collaboration with Northwestern University so that reporters could access automated backgrounders that would help them cover the 2020 presidential election in the United States. This figure has been reproduced from [ Diakopoulos *et al.*, 2020] with permission, under a CC-BY NC 4.0 license.

The online newspaper *El Confidencial* constitutes another example of the use of an internal self-editing tool. The data team set up previews that help journalists visualise the automated story they are about to generate, instead of having to work right off computer scripts. “We prepared a web tool that they could use [that includes] the new text [created] with a different condition, and in real time they can see (...) how the final article will look like”, said a technologist at the online newspaper. In another example of the use of automated backgrounders, the engineering team at the *Washington Post* and Northwestern University teamed up to create a query system that lets journalists access automated background information on the 2020 presidential election in the United States (see Figure 5): this contained for instance indications on the number and ethnic distribution of new registered voters in a given county ( Diakopoulos *et al.*, 2020; WashPost PR Blog, 2020a). As for automated notification streams, the BBC used Slack messages in a combined workflow to cover the 2019 general election in the United Kingdom with automated news (see Danzon-Chambaud, 2021c).

Finally, one last meaningful interference that could be observed has to do with generating output other than text, in this case NLG-to-audio content—which may come with the mediating effect of turning the written word into the spoken word. This was visible in the *Washington Post*’s and ABC’s efforts to create stories of their own, and not just audio content suited to help with vision impairment. Automated audio stories generated this way could then be tailored to a listener’s location as in the *Washington Post*’s election updates that were inserted in the newspaper’s political podcasts ( WashPost PR Blog, 2020b) or be accessed *via* virtual assistants (e.g., Amazon’s Alexa) as in the ABC’s diffusion of emergency alert summaries that were created using its own NLG tool ( Collett, 2021; Collett, 2022).

In this section, I highlighted how automated news is being used at 18 news organisations that were selected based on Hallin and Mancini’s (2004) media system typology (i.e., North Atlantic, North/Central and Mediterranean models) and that featured different media types (i.e., news agencies, newspapers, public service broadcasters). Using ANT, I demonstrated that force or effect passes through automated news in a linear way as initial intent is kept and when it does what it is supposed to do (i.e, *intermediary*), whereas in other cases there is substantial interference to it (i.e., *mediator*). The latter goes as follows: first, using alternate data sources while relying on a media organisation’s own internal feed, data collection or archives or, else, crowdsourced material or social media feeds; second, putting journalists at the centre of interfaces that are designed internally like in-house self-editing tools, notification streams and/or query systems for automated backgrounders; and, third, generating NLG-to-audio content as an output. These interferences, which are summarised in Table 2, are especially worth looking at since, according to Latour (2005, p. 128), “as soon as actors are treated not as intermediaries but as mediators, they render the movement of the social visible to the reader”.

**Table 2.** Automated news as *mediators*: areas with significant interferences, per media system.

NORTH ATLANTIC				
		News agencies	Newspapers	Public service broadcasters
***Sources***	Internal feed	–	–	ABC/BBC
	Own data collection	Reuters	–	–
	Archives	–	–	–
	Crowdsourced material	–	–	ABC
	Social media feeds	Reuters	–	–
***Interfaces***	In-house self-editing tool	Reuters	–	–
	Notification stream	Reuters	–	BBC
	Automated backgrounders	Reuters	Washington Post	–
***Outputs***	NLG-to-audio	–	Washington Post	ABC
NORTH/CENTRAL				
		News agencies	Newspapers	Public service broadcasters
***Sources***	Internal feed	–	–	–
	Own data collection	–	–	–
	Archives	STT	–	–
	Crowdsourced material	–	Stuttgarter Zeitung	–
	Social media feeds	–	–	–
***Interfaces***	In-house self-editing tool	–	–	–
	Notification stream	–	–	–
	Automated backgrounders	–	–	–
***Outputs***	NLG-to-audio	–	–	–
MEDITERRANEAN				
		News agencies	Newspapers	Public service broadcasters
***Sources***	Internal feed	–	–	–
	Own data collection	AFP	–	–
	Archives	–	–	–
	Crowdsourced material	–	–	–
	Social media feeds	–	–	RTVE
***Interfaces***	In-house self-editing tool	–	El Confidencial	–
	Notification stream	–	–	–
	Automated backgrounders	–	–	–
***Outputs***	NLG-to-audio	–	–	–

## Discussion and Conclusion

Using ANT’s lenses, considering automated news as *mediator* pointed out to significant interferences that are visible in the form of alternate data sources (i.e., own internal feed, data collection or archives, as well as crowdsourced material or social media feeds), interfaces that are designed internally and that allow for direct journalistic participation (i.e., self-editing tools, notification streams and/or query systems for automated backgrounders) and, finally, NLG-to-audio output. To paraphrase Latour (2005), this makes the movement of what can be considered the “actor-network of automated journalism” discernible to the researcher’s eye, giving it agency and thus answering RQ1. As for the potential implications that it bears for journalism practice and for journalism as a whole, it is perhaps necessary to break—so to speak—with one of ANT’s key tenets, which is keep any well-established sociological framework at bay. As a matter of fact, it has been argued that other sociological frameworks can be seen as “companion concepts”, which are encountered at a later stage of analysis ( Winthereik, 2020). Moreover, Couldry (2016, p. 5) stressed the importance of seeing ANT as “one important item in the media theorist’s toolkit that, like any tool, needs to be supplemented by others”.

I am then coming back to the work of Hallin and Mancini (2004), who based their media typology on the concept of *differentiation* and *de-differentiation*. Originally theorised by Parsons and Luhmann, differentiation assimilates modernity with “dividing society into stratified subsystems with specific specializations” ( West, 2021) while de-differentiation is about having these specialised structures return to a more homogeneous form. Following the logic of differentiation, Hallin and Mancini argued that the North Atlantic model of journalism sits the furthest away from social and political structures while the Mediterranean model presents strong ties between media and politics, which appear as two fields or sectors that often overlap. Finally, the North/Central European model is often situated somewhat in-between these two systems. They also observed that a process of de-differentiation driven by market forces seems to be steering the Mediterranean and North/Central European models further away from socio-political influences to bring them closer to the types of commercial values found in the North Atlantic model, resulting in making these media systems more homogenous.

Through this exploratory study we can start noticing a growing journalistic professionalisation in the way automated news is being employed, as it is drifting away from political and commercial influences (i.e., public service data, data brokers, automated content providers and third-party self-editing tools ^9^) to become more under journalists’ control, but also in citizens’ hand (i.e., using crowdsourced material as a source). As shown in Table 2, North Atlantic media organisations (i.e., Reuters, *The Washington Post*, ABC, BBC) seem to be leading the way in this process of *differentiation*, in accordance with Hallin and Mancini’s typology: as they write ( 2004, p. 80), “the Liberal Model is characterized by a high degree of differentiation of the media from other “other social bodies,” particularly those historically active in the political sphere”, which in this case also applies to techno-commercial influences. Hence, BBC’s and ABC’s use of internal feeds, Reuters’ own in-house self-editing tool and the *Washington Post*’s providing access to automated backgrounders—to name a few—all contribute to greater journalistic professionalisation by ensuring independence from all these forms of external influences. 

That being said, a process of *de-differentiation* could also be at play in that compliance with platforms’ terms and conditions is generally needed to be able to connect to social media APIs (see van Dijck *et al.*, 2018) and matching their technical standards is necessary to have automated audio stories featured on voice assistants (e.g., Amazon’s Alexa, Google Assistant). The question as to whether platforms or news organisations will act as *spokespersons* in this growing actor-network of automated journalism—and by extension RQ2—then remains open: should news media take on this role, for instance while developing their own self-editing solutions or relying on internal feeds, this could be interpreted as reinforcing the autonomy of the journalistic field, whereas—should they become too dependent on Big Tech companies for data acquisition and dissemination—this may result in making the field even more porous to techno-commercial influences. Interestingly, at the same time platforms are increasingly facing journalistic challenges while publishing content (e.g., fact-checking, neighbouring rights, etc.), which departs from their initial goal of connecting people or facilitating online search. Mutual *translations* may then well be on the way.

One limitation to this study has to do with a very much Western-centric selection of media organisations: at the time I reached out to interviewees, automated news was still a relatively new development that seemed to concern mostly news organisations based in the West, as well as some Asian newsrooms that I could not efficiently research because of my own language limitations. This meant I could not document the use of automated news in certain regions, like South America or East Asia. That being said, a growing number of scholars are now looking into these areas, among which figure research on the way automated news is employed at the Czech news agency ČTK ( Moravec *et al*., 2020) and across South American news media ( García-Perdomo *et al.*, 2022).

As mentioned in my methodology, another limitation relates to the impossibility of carrying out direct observations because of the COVID-19 pandemic. Although newsroom ethnography was initially considered—and even arranged for with a couple of newsrooms—these plans had to be cancelled when it became evident that the pandemic would last for longer than initially envisioned. Instead, I made use of remote semi-structured interviews and increased the number of news media under study. In consequence, I was not able to see how automated news was being used with my own eyes (although a virtual walkthrough was conducted with BBC): this resulted in me walking a fine line between findings I could directly document, like an automated news dashboard that is available online, and others that were reported or not directly visible to me, like details of a computer script. Even though I did my best to verify all of these elements, they are still exposed to the type of fallibility that goes with human interpretation. This is why I speak of an exploratory study, since more triangulation would be required to generalise these findings. 

One last limitation is about not being able to set in stone what remains essentially a field in flux, where new technical breakthroughs or ways of implementing automated journalism could be happening as I am writing these lines. For example, a couple of years ago, most NLG companies appeared to be external content providers only, in charge of creating automated news products in place of media companies, but then started offering self-editing tools as well, as in the case of Automated Insights (see Mullin, 2015). This fast-paced evolution of automated news products makes it difficult to analyse them based on development types (i.e., external content providers, in-house, third-party self-editing tools); however, this could be done once this is stabilised enough.

Other than this, possible research avenues include using ANT to determine whether, in the ongoing assemblage of an “automated journalism actor-network”, news organisations or platforms act as *spokespersons*, especially as it may turn into a *macro actor* able to restructure media production as a whole. To a certain extent, platforms can be seen as already gaining the upper hand as recent text summarisation efforts to create quizzes, polls or summaries—which are somehow related to automated news—appear to be quite tailored to fitting social media content. Such an analysis would be essential in determining power relationships likely to shape future developments of automated news products.

## Ethical approval

The Dublin City University Research Ethics Committee approved this study on the 25 ^th^ of February 2020, under the approval number: DCUREC/2020/032

## Figures and Tables

**Appendix A.** Details of interviewees’ news organisations, roles and gender as well as interview date and duration ^10^.

Interview	Organisation	Country	Position	Gender	Date	Duration
1	The Times	United Kingdom	Computational journalist	M	10/09/2020	00:44:15
2	Stuttgarter Zeitung	Germany	Editor	M	11/09/2020	00:49:28
3	France Bleu	France	Manager	M	07/10/2020	00:31:29
4	YLE	Finland	Senior technologist	M	08/10/2020	00:48:30
5	AP	United States	Executive	F	15/10/2020	00:30:12
6	BBC	United Kingdom	Manager	M	26/10/2020	00:42:53
7	NTB	Norway	Editor; Executive	M; M	19/11/2020	00:37:43
8	Tamedia	Switzerland	Senior computational journalist	M	20/11/2020	00:44:27
9	Bayerischer Rundfunk	Germany	Senior technologist	M	23/11/2020	00:35:27
10	Washington Post	United States	Executive	M	30/11/2020	00:29:21
11	STT	Finland	Executive	F	04/12/2020	00:31:09
12	Rossel/Sudpresse	Belgium/France	Executive	M	09/12/2020	00:44:21
13	El Confidencial	Spain	Executive; Technologist	M; F	15/12/2020	00:34:05
14	RTVE	Spain	Executive	M	16/12/2020	00:49:56
15	ABC	Australia	Manager	M	22/12/2020	00:45:09
16	BBC	United Kingdom	Senior technologist	M	22/03/2021	00:23:14
17	BBC	United Kingdom	Computational journalist	M	24/03/2021	00:41:21
18	BBC	United Kingdom	Journalist	M	29/03/2021	00:32:03
19	BBC	United Kingdom	Assistant editor	F	01/04/2021	00:12:11
20	ANSA	Italy	Executive	M	01/04/2021	00:21:21
21	BBC	United Kingdom	Technologist (1)	M	06/04/2021	00:42:01
22	AFP	France	Manager; Senior journalist	M; M	07/04/2021	00:34:52
23	BBC	United Kingdom	Technologist (2)	F	16/04/2021	00:33:00
24	Reuters	United Kingdom	Editor	M	20/04/2021	00:44:38
25	BBC	United Kingdom	Editor	M	28/04/2021	00:28:34

## Data Availability

Zenodo. Open Research Europe article "Automated news in practice": example of questionnaire 10.5281/zenodo.7953236 This project contains the following underlying data: Automated news in practice_ example of questionnaire.pdf (semi-structured questionnaire developed to interview an executive at The Washington Post.) Data are available under the terms of the Creative Commons Attribution 4.0 International license (CC-BY 4.0).

## Footnotes

1: At the time of writing, the New York Times reported that they and other outlets were shown a tool developed by Google, which is reportedly able to avoid the “pitfalls of generative A.I” ( Mullin & Grant, 2023). Yet there is little information available at the moment to verify this statement..

2: Except for a few exceptions, this article uses the same wording as my PhD thesis.

3: These “gatekeepers” could be, for instance, members of the marketing and advertising team, editorial managers or media executives.

4: There are a few shortcomings in this selection: first, Australia is not examined in Hallin and Mancini’s work for practical reasons, but they do specify that both Australia and New Zealand have close connections to Western European countries; second, Hallin and Mancini describe Belgium as a mixed case that sits between the Democratic Corporatist (i.e., North/Central) and the Polarised Pluralist (i.e., Mediterranean) models: in this study, I have included it under the latter category as Rossel and its subsidiary Sudpresse own titles in France and in French-speaking Belgium; third, there is the issue of limiting myself to Western news organisations, as discussed in my literature review and also in subsequent work on media systems (see Hallin & Mancini, 2011); finally, even if the Spanish newspaper *El Confidential* does not have a print edition, it defines itself as a “digital newspaper” ( *diario digital*), which is why it is included here.

5: The use of the term *translation* in ANT can be better understood when looking at its Latin roots: here, translation rather refers to *trans-latio*, which makes reference to a change in location ( Czarniawska, 2016).

6: For instance, Wu, Tandoc and Salmon (2019) used ANT to deconstruct the idea of seeing “automated journalism” as text generation only, arguing that media practitioners rather viewed it ( *ibid.*, p. 1453) as “anything from the machine aggregating and funnelling of content, to data scraping and auto-publication of stories”. Besides, ANT can also be used in student work on automated journalism, as in Falk Eriksen’s master thesis (2018) on the use of automated news by the Danish news agency Ritzau.

7: The data team at the Associated Press is also involved in setting up automated news for smaller scale projects, like polling results for each of the 50 American States or when transforming an exclusive dataset into local stories.

8: The Spanish public service broadcaster RTVE was also part of this initiative, mostly as an information provider, but is not mentioned here as it did not necessarily used automated news produced this way in a systematic manner.

9: Even if they can be limiting in terms of format or affordances (which cannot be tweaked internally), third-party providers or tools are interesting for news organisations that do not have the technical expertise in-house or are looking to use automated news for a one-off coverage, but not to maintain it in the long run.

10: Positions are based on my own understanding of interviewees’ roles and skills, and do not necessarily correspond to their official titles.
