# Supplementary material for: Automated news in practice: a cross-national exploratory study
Source: Open Res Eur. 2023 Nov 1;3:95. Originally published 2023 Jun 14. [Version 3] doi: 10.12688/openreseurope.16040.3 (PMC11008784; doi:10.12688/openreseurope.16040.3)
Supplement: Peer review history

# Reviewer report: Reviewer response for version 3

**DOI:** 10.21956/openreseurope.18148.r35996

**Published:** 2023-11-23

**Authors:** Nathalie Pignard-Cheynel, Andrew Robotham

**Affiliations:** 1 Academy of Journalism and Media, Faculty of Economics and Business, University of Neuchatel, Neuchatel, Switzerland; 2 Academy of Journalism and Media, Faculty of Economics and Business, University of Neuchâtel, Neuchâtel, Canton of Neuchâtel, Switzerland

The article “Automated news in practice: a cross-national exploratory study" by Samuel Danzon-Chambaud has satisfactorily addressed the remaining key points outlined in the previous round of revisions. Version 3 provides more clarity regarding data collection and analysis, as well a better contextualizing the relevance of Hallin and Mancini’s typology and (de)differentiation. As such it provides a worthy contribution to the field of journalism studies. This research provides both a good overview of the types of automated journalism tools being used across multiple countries and types of news media; something that fills a research gap at a time when such technologies are increasingly entering the mainstream. Furthermore, it offers several interesting insights into the underlying power-dynamics of automated journalism, and how these might evolve going forward.

 The extent to which automated journalism may vary from one media model/system (as defined by Hallin and Mancini) to another is a question that deserves further attention and investigation. This includes digging deeper for answers on how technologies such as automated journalism relate to different models/systems.

If applicable, is the statistical analysis and its interpretation appropriate?

Not applicable

Is the study design appropriate and is the work technically sound?

Partly

Is the work clearly and accurately presented and does it engage with the current literature?

Partly

Are the conclusions drawn adequately supported by the results?

Partly

Are sufficient details of methods and analysis provided to allow replication by others?

No

Are all the source data and materials underlying the results available?

Partly

Reviewer Expertise:

digital journalism, journalistic practices, information production systems, datajournalism

We confirm that we have read this submission and believe that we have an appropriate level of expertise to confirm that it is of an acceptable scientific standard.

# Reviewer report: Reviewer response for version 2

**DOI:** 10.21956/openreseurope.17910.r34938

**Published:** 2023-10-19

**Authors:** Julian Maitra

**Affiliations:** 1 Department of Communication and Media Research, University of Fribourg, Fribourg, Switzerland

The revised article titled "Automated news in practice: a cross-national exploratory study" by Samuel Danzon-Chambaud represents an original and important contribution to the study of news automation in journalism. The revision addressed the major concerns raised in the first review round. It is now clear that the study uses an exploratory approach to assess the current state of automated news across 18 news publishers in Europe, North America, and Australia. 

 The study's methodological combination of Hallin and Mancini's media system typology and actor-network theory (ANT), a science, technology, and society approach popularized by researchers such as the French sociologist Bruno Latour, makes it an exciting addition to the journalism studies literature on automated journalism. ANT is particularly well suited to take into account the increasingly strong non-human agency technologies, such as news automation, assumed in the complex socio-technological networks where contemporary newsrooms operate.

 Based on the clarifications and substantive improvements applied by the author in the revised manuscript, I can recommend this study for indexing without further modifications.

If applicable, is the statistical analysis and its interpretation appropriate?

Not applicable

Is the study design appropriate and is the work technically sound?

Partly

Is the work clearly and accurately presented and does it engage with the current literature?

Yes

Are the conclusions drawn adequately supported by the results?

Partly

Are sufficient details of methods and analysis provided to allow replication by others?

Partly

Are all the source data and materials underlying the results available?

Partly

Reviewer Expertise:

digital journalism, news audiences, media sociology

I confirm that I have read this submission and believe that I have an appropriate level of expertise to confirm that it is of an acceptable scientific standard.

# Reviewer report: Reviewer response for version 2

**DOI:** 10.21956/openreseurope.17910.r34939

**Published:** 2023-09-28

**Authors:** Nathalie Pignard-Cheynel, Andrew Robotham

**Affiliations:** 1 Academy of Journalism and Media, Faculty of Economics and Business, University of Neuchatel, Neuchatel, Switzerland; 2 Academy of Journalism and Media, Faculty of Economics and Business, University of Neuchâtel, Neuchâtel, Canton of Neuchâtel, Switzerland

The changes made to the article are satisfactory overall. The main comments from the first round of reviewing have indeed been addressed. In particular, the framing of the article as an exploratory study rather than case studies (point 2.1 below), and fits with the overall research method and data used. Furthermore, several improvements have been made to how ANT was presented (3.1-3.3; 4.1). However, we still believe that further information is needed regarding how data was analyzed (1.2-1.4). Lastly, we remain wary of the reference to Hallin and Mancini's model, especially in view of the claims that suggest some kind of causal link has been established (1.2; 2.2).

**Point for improvement 1 from the first round of reviewing: *The paper lacks important details as to how the data was analyzed.***

**Points improved:**

 1.1 Version 2 shows several improvements as to how the data were analyzed.

**Recommended further improvements:**

 1.2 A brief paragraph answering the following questions is required to shed light on some of the method-related questions that remain unanswered to date:

- Once collected, how was the interview material treated, classified (for example into those categories outlined in table 2), analyzed, etc.?
- What role did your ANT framework play at this stage of data analysis?
- In particular, which (objective) criteria were used to make the distinction between linear and non-linear?

This is important because in the absence of such details, the article may give the impression of a confirmation bias towards fitting the results to the Hallin and Mancini model.

 1.3 Further clarification is also required because the author does not discuss what degree his semi-structured interviews were used to collect the experiences, opinions, journalistic roles and perceived realities (related to automated journalism) of their interviewees on the one hand, and used to recreate a factual description or “model” of the technologies discussed on the other. Beyond being merely a practical matter, it is an epistemological one, since ANT - and especially the Latourian kind - is unique in the way it argues in favor of bringing these two perspectives together (see Latour, 2005, 87-99). The interview guide provided contains questions pertaining to both levels. Yet, it is unclear if/how the former were used. To put it differently, one of the keys to a successful ANT study is overcoming this “artificial” distinction and to explain how the human and non-human are intertwined, and to what effect.

 1.4 It would be helpful if the author contextualized the interview data within the broader scope of the PhD research project. The disclosure is welcome, but practical implications should be addressed briefly. Did all the questions of the interview guide pertain to this study only or were they used in view of further research? This will help clarify the previous point.

**Point for improvement 2 from the first round of reviewing: *Despite the large number of interviews carried out, most of the media are based on a single interview, which may prevent generalization and, in any case, does not allow us to claim the term "case study".***

**The main issue has been addressed:**

 2.1 The use of the term exploratory instead of case study is welcome and better reflects the nature of the study. Indeed, the sampling strategy outlined in the paper now makes perfect sense and provides some great insights.

**Recommended further improvements:**

 2.2 Regarding Hallin and Mancini’s classification and the issue of differentiation, we are wary of the assertion regarding the link between differentiation and the North Atlantic model on the basis of the author’s purposive sampling. Thus, we believe this should be framed as an interesting avenue for future research, rather than a causal relationship that has been established through solid empirical investigation.

**Point for improvement 3 from the first round of reviewing: *ANT was doubtlessly a key resource in designing the project and certainly played a role in how the project was designed and the data analyzed and interpreted. However, the prominence of ANT theory and terminology obscures the key takeaways and does not, in our opinion, contribute to a better understanding of automated news for the (average) reader.***

**The main issues have been addressed:**

 3.1 The additional references to specific media-related ANT research are welcome.

 3.2 The shift from intermediary vs mediator to linear vs interferences does, in our opinion, help make the article more accessible. The point related to how interferences are, in fact, expressions of (or traces of) journalistic autonomy is interesting and well-argued. It is much clearer in the revised version.

 3.3 On the other hand, the addition in the conclusion section of the concept of enrolment is welcome and contributes to better understanding what is at stake regarding the (successful) adoption of automated journalism.

**Recommended further improvements:**

 3.4 The changes add new terms concepts to an already long list of related to so-called ANT infralanguage. Many of these terms are introduced in the “ANT Analytical Framework” section, without being used in the results section or the conclusion (introduced but not used: *obligatory point of passage, heterogenous entities, simplification, black-boxing and punctualisation, immutable mobiles*). While this shows that the author has a good theoretical understanding of ANT, it doesn’t necessarily benefit the article. For example, the space used for presenting ANT in general terms (which has been done many times and could easily be referred to with a single reference), may be better used to provide a more technical explanation of how their identify interferences, and how and why these related to mediators and differentiation.

**Point for improvement 4 from the first round of reviewing: *At the very least, we would recommend putting less emphasis on ANT and restructuring the results section according to types of automated news and their uses (intended or not). This would also free up space to contextualize more, engage more with existing literature (about automated news) and better define the concepts and research questions. The addition of a specific "Discussion" section would be relevant.***

**The main issues have been addressed.**

 4.1 Except for minor reservations expressed in the point above, this issue has been adequately addressed.

If applicable, is the statistical analysis and its interpretation appropriate?

Not applicable

Is the study design appropriate and is the work technically sound?

Partly

Is the work clearly and accurately presented and does it engage with the current literature?

Partly

Are the conclusions drawn adequately supported by the results?

Partly

Are sufficient details of methods and analysis provided to allow replication by others?

No

Are all the source data and materials underlying the results available?

Partly

Reviewer Expertise:

digital journalism, journalistic practices, information production systems, datajournalism

We confirm that we have read this submission and believe that we have an appropriate level of expertise to confirm that it is of an acceptable scientific standard, however we have significant reservations, as outlined above.

# Response

**Published:** 2023-10-26

**Authors:** Samuel Danzon-Chambaud

Dear Prof. Pignard-Cheynel and Dr. Robotham,  

 Thank you for your additional feedback on this article, which helped me clarify the points you raised. Please find below how I addressed them:  

 I have specified the context in which the interviews took place. They were indeed done as part of the larger PhD project, and so the model questionnaire contains descriptive questions as well as more interpretative ones. This is now mentioned in Methods, at the end of the third paragraph.  

 This also helps explain how I went on with my data analysis, focusing on descriptive elements and sorting them using ANT's *linear* (i.e., *intermediaries*) vs. *interference* (i.e., *mediators*) effect. I have added a short explanation in the paragraph under Results.  

 As for the criteria that were used to make this distinction, they are each specified in the first sentence(s) that follow “Linear situations” and “Interferences”, in the Results section.  

 I have followed your advice on being cautious about causal relationship with Hallin and Mancini’s framework. This is why I now caution against this at the beginning of the second paragraph of the Discussion/Conclusion, specifying meanwhile that it could provide room for future research.  

 Lastly, I have taken out from the ANT section “infra-language” that was not used in the rest of the article. Peer-reviewed summaries of ANT (i.e., Crawford, 2005; Nikolova, 2008; Cooren, 2009; Hassard & Alcadipani, 2010; Czarniawska, 2016; Michael, 2027a, 2017b; Blok, 2019; Primo, 2019) are referenced throughout the ANT framework part, should the reader want to read more about it.   

 I hope these modifications are in line with your expectations, and that they make the article fit for publication.  

 Yours sincerely, Samuel Danzon-Chambaud

# Reviewer report: Reviewer response for version 1

**DOI:** 10.21956/openreseurope.17320.r33320

**Published:** 2023-07-24

**Authors:** Julian Maitra

**Affiliations:** 1 Department of Communication and Media Research, University of Fribourg, Fribourg, Switzerland

**Summary:**

- The study addresses a highly relevant topic in journalism studies: the increasing adoption of news automation technology across media systems and newsrooms. The study’s innovative research design combines empirical and theoretical approaches. On the one hand, the author conducted 18 semi-structured interviews with news professionals from 11 Western countries between 2020 and 2021. The interviewees included employees from news agencies (e.g., AFP), public service broadcasters (e.g., BBC), and newspapers (e.g., Stuttgarter Zeitung). Additional empirical insights were generated through a news “netnography”.
- On the other hand, the author applied a theoretical lens to contextualize the empirical findings. This lens is inspired by actor-network theory (ANT), Hallin and Mancini’s media system typology (2004), and sociological notions of professionalization.
- A key finding using ANT terminology is that news automation technologies have been adopted in two distinct ways: First, as *intermediaries* of journalistic work. Such “predictable” use may include cases where “private or public service datasets are being used as sources, when there is no journalistic involvement other than through the affordances already provided for by third-party tools” (p. 7). For example, the AP cooperated with the NLG firm Automated Insights, to provide template-based software to automate corporate earnings reports.
- Second, the author identified cases of a more “transformative” adoption of news automation when the technology *mediates* journalistic work. Such use of news automation is described as “drifting away from political and commercial influences [e.g., from third-party tech platforms and NLG firms]… to become more under journalists’ control” (p. 13). This occurred, for example, when the BBC autonomously developed in-house news automation technology that relied on its proprietary data (and not on third-party data and platforms).
- The study argues that such a mediating adoption of news automation translates into higher professional autonomy for news organizations. The study finds that such *differentiation* (another ANT concept) is particularly prevalent among news organizations from the North Atlantic media system. Nevertheless, *dedifferentiation* processes may also occur in the actor-network, for example, when journalistic actors grow more dependent on third-party platforms for access to mediating news automation technology and data.

**Major review points:**

- **The study could be improved through a more coherent theoretical focus that explains the empirical findings better**. The study currently employs elements of ANT, Hallin and Mancini’s media system typology, the sociology of professions, and sociological field theory (for the latter, see, e.g., p. 13). Each of these frameworks has its merits in journalism studies, particularly in the context of explaining issues of digital innovation. However, in the present study, it is less clear how the theory adds explanatory value to the empirical findings. ANT, for example, could be better used to explain the interrelationships in the actor-network of news automation in the analyzed newsrooms. How do the complex assemblages and interdependent agencies of journalistic actors, news stories, news automation algorithms, databases, tech platforms, etc., interrelate? In contrast, Hallin and Mancini’s comparative framework could be more convincingly used to explain the reasons behind the different uses of news automation across media systems. Perhaps it could also be a solution to focus on one theoretical framework and provide a deeper explanation of its implications.
- **Clarify the data collection through semi-structured interviews**: The provided exemplary interview questions, which were specifically designed to interview a news professional from the Washington Post, raise questions about how the data was collected. Were all interviewees asked the same (or very similar) questions, or were they tailored to each respondent, as in the example? It would no longer constitute a semi-structured interview approach in the latter case. The author should clarify the data collection method to justify the representativeness of the empirical insights.
- **Unconvincing generalization of empirical (case study) findings**: While the empirical insights gained in the 18 interviews are valuable, they represent case study observations from specific organizations at specific points in time. The author used “purposive” – i.e., subjective – sampling to construct the dataset (p. 5). Therefore, some claims in the conclusion section, such as “All in all, this testifies of a growing journalistic professionalization in the way automated news is being employed, as it is drifting away from political and commercial influence…” (p.13), seem problematic. Likewise, concluding that news organizations from the North Atlantic media system are more adept at using news automation based on the interview insights from three news organizations (WaPo, ABC, BBC) is also not convincing. Such generalizations cannot be made without comprehensive data collection across much larger samples or through the use of statistical techniques of random sampling. While the author acknowledges some limitations, i.e., that the “field is in flux” (p. 13), the study could be improved by better clarifying how and to what extent its findings are generalizable.

**Minor points:**

- **Hard to read sentences**: While the paper is generally well-written, several sentences are too long and sometimes ambiguous, making their meaning difficult to decipher. The clarity of the writing could be improved by using topic sentences at the beginning of paragraphs and by limiting sentences to ca. 25 words. For example, consider breaking up the following one-sentence-paragraph with 135 words (p.9) into multiple sentences:
  "Another area where additional translations are brought into force relates to automated news systems that are specifically built for journalists, other than through the affordances already provided for by third-party self-editing tools. These can be, first, internal software that comes with its own self-editing tool, features notification streams and provides access to auto-generated background information, as in Reuters’ Lynx Insight system where journalists can template out their own stories using a form of No-code language that resembles those of third-party tools (see Figure 4), receive Microsoft Teams notifications once stories generated this way or that the data team set up are ready and query this system as they look for automated news with background information on the subject that they are covering."
- Similarly, the following sentence on p.10 is too long and hard to understand:
  "Using ANT, I demonstrated that some of them used automated news as intermediaries, where initial intent is kept and where it does what it is supposed to do, while others considered it more as mediators while bringing in additional meaningful translations, which go as follows: first, enrolling alternate data sources while relying on a media organisation’s own internal feed, data collection or archives as a source or, else, on crowdsourced material or social media feeds; second, enticing journalists while putting them at the centre of interfaces that are designed internally like in-house self-editing tools, notification streams and/or search features to access automated backgrounders; and, third, enlisting vocal elements through generating NLG-to-audio content as an output."
- **Unclear explanatory value of screenshots**: It remains unclear how some figures (e.g., Figures 3 and 5) contribute to or exemplify the findings of the study. The author should better explain what they show and how this sustains his arguments.
- **Interview date accuracy**: Appendix A mentions two interviews (in Norway and Switzerland) were conducted in 2010. Is this information correct?

If applicable, is the statistical analysis and its interpretation appropriate?

Not applicable

Is the study design appropriate and is the work technically sound?

Partly

Is the work clearly and accurately presented and does it engage with the current literature?

Yes

Are the conclusions drawn adequately supported by the results?

Partly

Are sufficient details of methods and analysis provided to allow replication by others?

Partly

Are all the source data and materials underlying the results available?

Partly

Reviewer Expertise:

digital journalism, news audiences, media sociology

I confirm that I have read this submission and believe that I have an appropriate level of expertise to confirm that it is of an acceptable scientific standard, however I have significant reservations, as outlined above.

# Response

**Published:** 2023-09-06

**Authors:** Samuel Danzon-Chambaud

Dear Dr. Maitra,  

 Thank you for providing me with helpful feedback, which helped make this work better and clearer. Please find below how I addressed your comments:

 The theoretical focus has been reworked to put less emphasis on ANT. At the same time, I also tried to better detail how this theoretical framework helped inform my data analysis. I paid special attention to better explaining how I worked with the concept of *intermediaries* and *mediators*, relying for that on an analogy with *linear* usages and substantial *interferences* to it.  

 It is true that how automated news connects to the wider network has not been part of this analysis. However, seeing it as *mediator* (i.e., when interference is made to linear usage)—as this study strives to—opens up new research possibilities for tracing these connections. Although I took it out not to overload the conclusion, this relates to Latour’s belief that “there exist translations between mediators that may generate traceable associations”.  

 A brief hint as to what these future translations/negotiations may imply is given at the end of the article: as there is substantial interference to platforms’ linear way of working when playing a journalistic role (making them mediators), mutual negotiations are then likely to happen with automated news.           

 My use of Hallin and Mancini has also been reworked for clarity’s sake. In the Methods section I explain how my news organisation selection has been inspired by it, leaving the theoretical discussion on differentiation and de-differentiation to the Discussion/Conclusion part. That way, it is better integrated with my analysis of journalistic autonomy.     

 My use of semi-structured interviews has been clarified in the Methods section: I stress that I used essentially the same structure, except for the bit of flexibility that was required for each interview.  

 The point you made about generalisation is an important one. I have added a paragraph at the end that details the limitations I faced, especially given that I was not able to conduct investigations on the ground (because of COVID-19). I am being forthcoming about this, and explain how this impacts the generalisation of my research findings. This is why I now speak of an *exploratory* study.   

 I also rewrote hard-to-understand sentences and fixed the mistakes you spotted. The screenshots are now part of what I describe as supplementary informative material, as I took out the term “netnography” (which implied a greater role than what I envisioned).  

 I hope that you see these changes as adequate, and that this new version meets your expectations.  

 Yours sincerely, Samuel Danzon-Chambaud

# Reviewer report: Reviewer response for version 1

**DOI:** 10.21956/openreseurope.17320.r33205

**Published:** 2023-07-17

**Authors:** Nathalie Pignard-Cheynel, Andrew Robotham

**Affiliations:** 1 Academy of Journalism and Media, Faculty of Economics and Business, University of Neuchatel, Neuchatel, Switzerland; 2 Academy of Journalism and Media, Faculty of Economics and Business, University of Neuchâtel, Neuchâtel, Canton of Neuchâtel, Switzerland

**Strengths of article:**

- This article deals with a highly relevant topic for the media, research and society in general.
- The research questions, which would benefit from being reformulated, are interesting. They deserve to be better contextualized.
- The empirical work is impressive, both in terms of the number of media studied and the diversity of countries represented. The dataset allows for an important contribution to journalism studies, in spite of some methodological limitations.
- The results are interesting and contribute to research on the topic.
- The article is well-written and clearly structured.

**Points for improvement:**

- The paper lacks important details as to how the data was analyzed.
- Despite the large number of interviews carried out, most of the media are based on a single interview, which may prevent generalization and in any case does not allow us to claim the term "case study".
- ANT was doubtlessly a key resource in designing the project and certainly played a role in how the project was designed and the data analyzed and interpreted. However, the prominence of ANT theory and terminology obscures the key takeaways and does not, in our opinion, contribute to a better understanding of automated news for the (average) reader.
- At the very least, we would recommend putting less emphasis on ANT and restructuring the results section according to types of automated news and their uses (intended or not). This would also free up space to contextualize more, engage more with existing literature (about automated news) and better define the concepts and research questions. The addition of a specific "Discussion" section would be relevant.

**General context, topic and research questions:**

 The article’s overall topic of automated news is relevant to the field of journalism studies and addresses questions that have become of increasing interest to scholars and industry over the past couple of years (in other words since the field data was collected) and especially over the past year, notably with the advent of ChatGPT, which has elevated questions about news algorithms and AI to become some of the most pressing questions in many newsrooms. Such questions have become key strategic considerations in many newsrooms, not just those more innovative news media for which data has been collected for this study. Although the recent context cannot be addressed with published peer-reviewed research (no doubt there is a new wave of research underway that will begin to be published over the coming months/years), it can nevertheless be discussed in other ways, thereby making your research more relevant to readers. Although this could hardly be considered a weakness of the paper in its current form, it seems to us that there is a missed opportunity.

 The introduction could situate the core problem(s) more precisely, and from the outset. There is a leap from the topic to the very specific research questions (addressed below) on the back of the presentation of the *ANT theoretical perspective*. Which journalistic and societal issues is this research related to and why? Why do we need an answer and how might an answer contribute to the research field, to industry and to society? Who has tried to answer these broad questions before? This overarching context requires some work.

 Regarding the research questions themselves and their formulation:

- *“RQ1: Using ANT’s lenses, what is the overall direction that automated news systems seem to be taking?”*
  It does not seem like you have defined “overall direction”. It should be avoided using a term such as “seem” in a research question. In its current formulation it is of course a matter of guesswork, which cannot be evaluated scientifically. However, a simple reformulation is probably sufficient.
- *“RQ2. What are the implications of these developments for journalistic professionalisation?”* This research question is interesting but might benefit from clarification or reformulation (either by being better introduced above, or by being slightly reformulated). It is unclear from which viewpoint you are examining these implications (and the underlying theoretical assumptions). Are we interested in how we think journalists (or news professionals) expect journalistic professionalization related to automated news to develop and evolve, based on their discourses? Are we trying to document current practices, with a view to gaining a better understanding of how things might evolve?

**Literature and theoretical concepts:**

 The literature review is well written (as is most of the article) and includes much of the key research on the topic of automated news. However, some of the literature on automated news/algorithmic news is a little dated and we would recommend the inclusion of some more recent work. The references are good and certainly warrant inclusion, but a lot has happened since 2015 (there are some references to literature in the results section that may be worthy of mention earlier on). The author mostly uses the literature to provide a history of the use of automated news but provides little in terms of the results and insights of previous research. The context is important, but the author could engage more with the results of previous research. In addition, the works cited deserve to be better integrated into a problematization effort, rather than simply summarizing the state of the art on the issue.

 The ANT theoretical perspective is very well researched and presented (it is often not). The author refers to the seminal works of ANT and does well in explaining the key concepts. Since it is a perspective that has been used quite a lot in journalism and media studies (works that should be cited in the article), it might make sense to focus a little more on the more specific literature, and to introduce those media and journalism studies that have used ANT to study tools and technologies most similar to the research topic.

 The author refers to a broad range of ANT concepts, or rather terms that Latour refers to as “infra-language”. Yet, the concepts referred to in the analysis/results are (mostly) limited to mediators vs intermediary, and translation (often without the process of translation being described in detail). Thus, we are skeptical of the prominence of ANT in the study, and would not recommend using it in the title. Further methodological points related to ANT are discussed below.

 In view of their prominence in the introduction and the conclusion, we would find a brief explanation of the concepts of differentiation and dedifferentiation helpful (beyond the broad elements presented in the footnote).

**Research design:**

 The research design – case studies of the use of automated news by eight newsrooms in different countries – is ambitious.

 The author deserves praise for having managed to access news professionals in such a wide range of countries and types of media. The use of Hallin and Mancini’s media system typology is certainly defendable. However, because it only includes one media per category, it should be argued that the sampling method offers a strategy to access a very wide range of phenomena and uses of automated news, rather than allowing for any significant comparisons between the media systems in question. The dataset of 25 semi-structured interviews from 18 news organizations is impressive. However, only the BBC includes more than one interview (8 interviews). The claim to have conducted multiple case studies based on a single research interview (with the exception of the eight conducted with the BBC) is a bold one. This is not to say that the research is not valid, but since the reference for case study research is Yin (1994), we would claim that this research does not match the criteria for a case study. Using this term in the title and several times in the text is therefore problematic.

 The use of a combination of semi-structured interviews and complementary “netnographical” research is appropriate and certainly not incompatible with the research questions. However, the limitations of the method and data collected should be outlined more clearly. For example, semi-structured interviews are not best-suited for accessing an objective reality that is “out-there”, but are more appropriate for constructivist perspectives, or require triangulation.

 It is not clear how either type of data is analyzed (nor how much “netnography” was conducted, nor is it clear how the ANT perspective is concretely applied, despite the clear introduction to ANT (discussed below).

 One of the criticisms of ANT is that it claims to be more method than theory, while providing little help in terms of practical implementation. However, it would have been helpful to explain how this analytical lens was used. One would not know where to begin if asked to replicate the study on a different dataset. From the outside, it mainly consists of the distinction between mediators and intermediaries, based on how the automated content is used, and/or reclaimed for other purposes. This is an extremely interesting question. And although we are certain that the ANT literature was key in obtaining some of the insights, we fear that the ANT “infra-language” might in fact render the results and discussion less clear. Since Bruno Latour’s *Reassembling the social* is cited, it should also be noted that the approach is described as the painstaking reconstruction of complex links and associations between entities. Such depth would require multiple interviews for each case and an in-depth analysis of digital tools and technologies, for which, as stated, we have little methodological information and do not have access to the dataset (see below), and which is described as complementary.

 Despite an in-depth knowledge of ANT literature, we were unable to understand several elements of the results section. This raises suspicions as to the author’s own mastery of ANT. On the other hand, it is possible that the points being made are much simpler than they appear and as such, would benefit from much simpler language. In short, the research design and data gathered provide many useful insights into automated news, but it is not necessarily what it claims to be (i.e., 18 ANT case studies of automated news).

**Results and conclusions:**

 The information provided in the results section is precious, since it gathers a large number and wide range of technologies that can be defined as “automated news”, and related uses. As discussed previously, we are not certain that using ANT concepts makes for a better reader experience. In our view, classifying the different types of automated news and their uses – intended and unintended – would make for a better article. To do this, it would be necessary to confront the results obtained with the rich literature on automated news and the use of artificial intelligence in journalism. In this way, the article will find a better place in relation to the existing literature.

 There are several interesting points made in the conclusion (e.g. internalizing vs Big tech). However, they a lost among the complexity of the ANT interpretations. To journalism scholars and researchers alike, framing the results from the perspective of what is at stake for journalism (and by extension for society at large) would be much more interesting and helpful.

**Data:**

 Consisting mostly of semi-structured interviews with news professionals, the absence of source data is understandable. The examples, tables and screenshots facilitate understanding for a topic that can be quite abstract. However, unlike the interview, data, we suspect that at least some of the netnographical data could have been made available (notwithstanding business secrets). In the absence of such material, a simple list of the data analyzed would have provided better transparency.

If applicable, is the statistical analysis and its interpretation appropriate?

Not applicable

Is the study design appropriate and is the work technically sound?

Partly

Is the work clearly and accurately presented and does it engage with the current literature?

Partly

Are the conclusions drawn adequately supported by the results?

Partly

Are sufficient details of methods and analysis provided to allow replication by others?

No

Are all the source data and materials underlying the results available?

Partly

Reviewer Expertise:

digital journalism, journalistic practices, information production systems, datajournalism

We confirm that we have read this submission and believe that we have an appropriate level of expertise to confirm that it is of an acceptable scientific standard, however we have significant reservations, as outlined above.

# Response

**Published:** 2023-09-06

**Authors:** Samuel Danzon-Chambaud

Dear Prof. Pignard-Cheynel and Dr. Robotham,  

 Thank you for carefully reviewing my work, and for your detailed feedback. Please find below how I addressed your recommendations, which helped make this article stronger:  

 I have put less emphasis on the use of ANT, while also detailing a bit better how this theoretical framework informed my data analysis. Instead of “intermediaries” I now speak of instances where automated news is used in a *linear *way, and instead of “mediators” I refer to situations where substantial *interference* to it is made. The aim here is—as you wrote—to limit the use of ANT’s “infra-language” so as to make the text more readable.  

 I have reworked a bit my general overview of ANT so that some concepts are clearer (i.e., the more “passive” role of intermediaries as opposed to the more “active” contribution of mediators). I also mention some scholarly work that use ANT as a framework to study journalism practice.    

 As you suggested, I have provided a brief explanation of how algorithms are used in news production (e.g., investigative reporting, fact-checking), and better detailed what is at stake when studying automated news. I have rephrased my research questions accordingly, stressing that automated news should be rather studied when yielding agency.    

 I also briefly touch on generative AI (ChatGPT, Bard) in the Introduction, explaining that at this stage I believe its opacity and unreliability does not make for journalistic usage.  

 I can see how the use of the term “case study” is problematic. Instead, I now speak of an *exploratory* study and updated the text and title accordingly.  

 Likewise, the use of the term “netnography” implies a much greater role than what I envisioned. I am now referring to informative material that complement my research interviews.                 Limitations to using research interviews as a means of data collection are articulated more clearly at the end of the article, including the need for additional input to be able to generalise.    Because of the length of the article, I have kept the brief definition of differentiation and de-differentiation theory as-is (I only added that it originates from the work of Parsons and Luhmann). However, I have moved it to a Discussion/Conclusion part where it is better integrated with my analysis of journalistic autonomy.  

 I hope that these changes meet your expectations, and that you see this modified version as a worthful contribution to journalism studies.  

 Yours sincerely, Samuel Danzon-Chambaud
